# Dual-stream disentangled model for microvascular extraction in five datasets from multiple OCTA instruments

**DOI:** 10.3389/fmed.2025.1542737

**Published:** 2025-01-29

**Authors:** Xiaoyang Hu, Jinkui Hao, Quanyong Yi, Yitian Zhao, Jiong Zhang

**Affiliations:** ^1^Faculty of Electrical Engineering and Computer Science, Ningbo University, Ningbo, China; ^2^Laboratory of Advanced Theranostic Materials and Technology, Ningbo Institute of Materials Technology and Engineering, Chinese Academy of Sciences, Ningbo, China; ^3^Ningbo Eye Hospital, Wenzhou Medical University, Ningbo, China

**Keywords:** OCTA, cross-instruments, microvascular segmentation, vessel measurements, disentanglement

## Abstract

**Introduction:**

Optical Coherence Tomography Angiography (OCTA) is a cutting-edge imaging technique that captures retinal capillaries at micrometer resolution using optical instrument. Accurate segmentation of retinal vasculature is essential for eye related diseases measurement and diagnosis. However, noise and artifacts from different imaging instruments can interfere with segmentation, and most existing deep learning models struggle with segmenting small vessels and capturing low-dimensional structural information. These challenges typically results in less precise segmentation performance.

**Methods:**

Therefore, we propose a novel and robust Dual-stream Disentangled Network (D2Net) for retinal OCTA microvascular segmentation. Specifically, the D2Net includes a dual-stream encoder that separately learns image artifacts and latent vascular features. By introducing vascular structure as a prior constraint and constructing auxiliary information, the network achieves disentangled representation learning, effectively minimizing the interference of noise and artifacts. The introduced vascular structure prior includes low-dimensional neighborhood energy from the Distance Correlation Energy (DCE) module, which helps to better perceive the structural information of continuous vessels.

**Results and discussion:**

To precisely evaluate our method on small vessels, we delicately establish OCTA microvascular labels by performing comprehensive and detailed annotations on the FOCA dataset, which includes data collected from different instruments, and evaluated the proposed D2Net effectively mitigates the challenges of microvasculature region recognition caused by noise and artifacts. The method achieves more refined segmentation performance. In addition, we validated the performance of D2Net on four OCTA datasets (OCTA-500, ROSE-O, ROSE-Z, and ROSE-H) acquired using different instruments, demonstrating its robustness and generalization capabilities in retinal vessel segmentation compared to other state-of-the-art methods.

## 1 Introduction

Optical Coherence Tomography Angiography (OCTA) is a non-invasive imaging modality (1) that provides high-resolution, three-dimensional images (2). Unlike fundus photography, which lacks detailed microvascular information, and fluorescein angiography (FA) (3), which may have negative effects on human subjects (4), OCTA imaging offers a safer alternative by providing rich retinal microvascular visualizations (5, 6). Consequently, it has been widely accepted and utilized in clinical practice for retinal vascular imaging (7). However, due to the presence of significant independent noise and artifacts in three-dimensional scan data, it is common practice to project 3D image data onto a two-dimensional *en face* image for analysis (4). The *en face* images are categorized into three different complexes: the Inner Vascular Complex (IVC), the Superficial Vascular Complex (SVC), and the Deep Vascular Complex (DVC) (8).

Changes in retinal vascular morphology can be used not only for the clinical diagnosis of ocular diseases (9) but also for analyzing the severity of systemic diseases and evaluating the effectiveness of treatments (10). Previous research has revealed a significant correlation between abnormal OCTA retinal morphology and numerous diseases (4), such as early glaucomatous optic neuropathy (11, 12), diabetic retinopathy (13–15), age-related macular degeneration (16, 17), and Alzheimer's disease (18, 19). Consequently, quantifying retinal vascular biomarkers holds paramount clinical importance (20).

**Figure 1:**
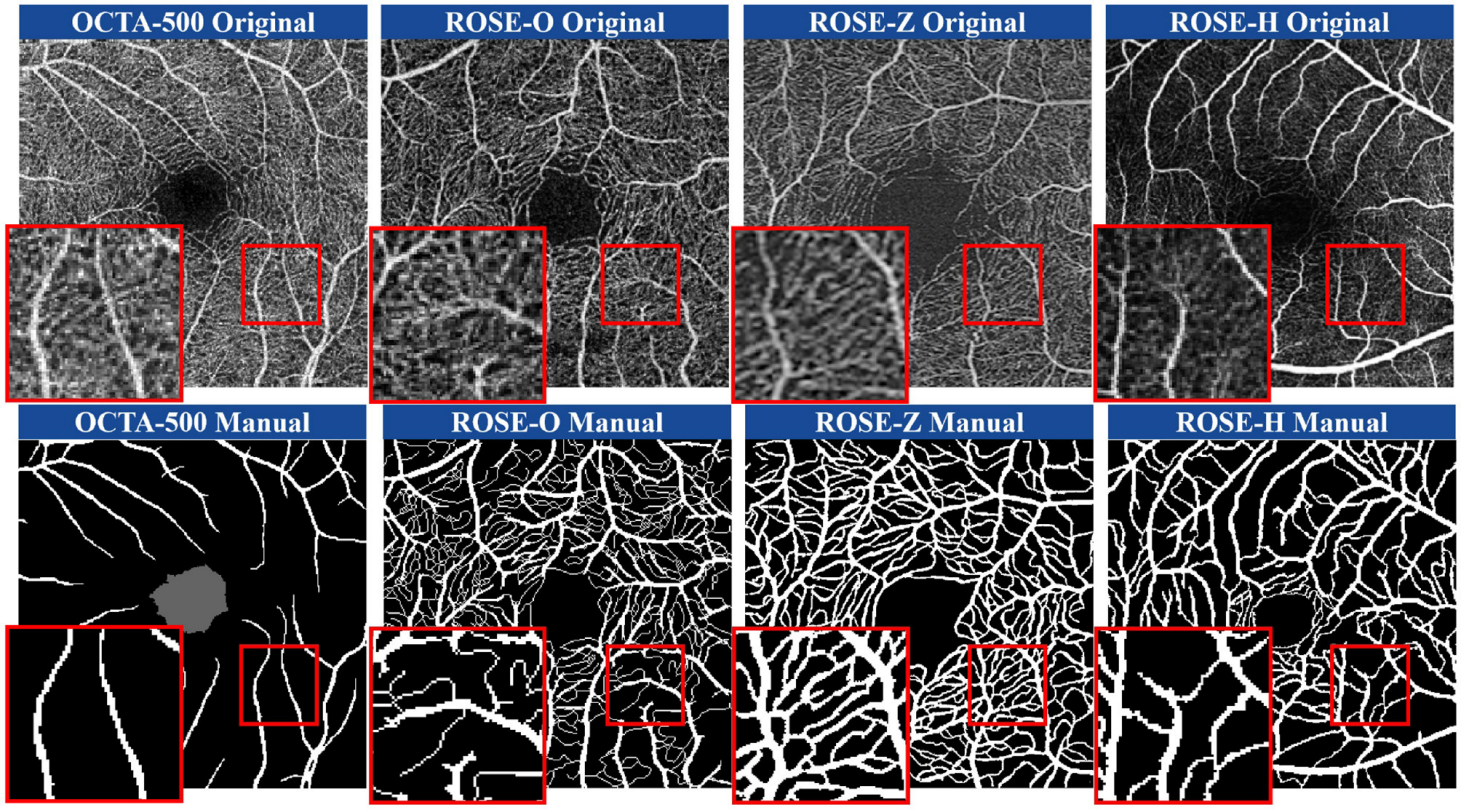
Illustration of SVC layer scans in four different datasets obtained from multiple instruments and the corresponding manual annotations. From left to right: OCTA-500, ROSE-O, ROSE-Z, ROSE-H.

With the success of deep learning algorithms, many methods have been developed to address the vascular extraction in OCTA images (21, 22). However, publicly available datasets primarily focus on the segmentation of large blood vessels. Figure 1 illustrates the SVC scans of the OCTA-500 (23), ROSE-O (4), ROSE-Z (8), and ROSE-H (8) datasets, along with their corresponding manual annotations. In the magnified views, we can observe rich microvascular structures in both datasets. However, these microvascular details have not been completely annotated. Thus, the primary goal of our method is to achieve precise segmentation of finer microvascular structures.

**Figure 2:**
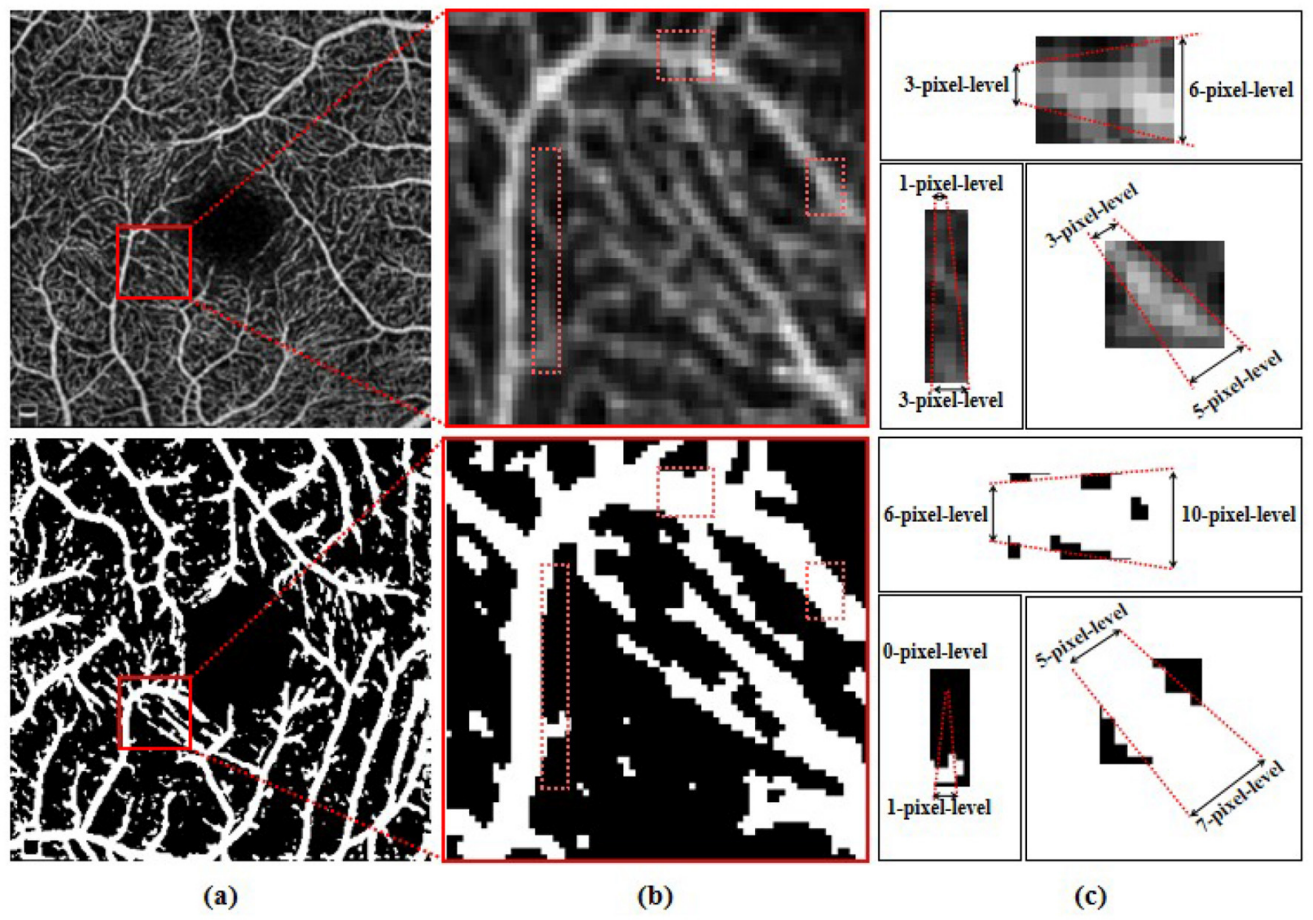
**(A)** Original OCTA image and the schematic representations of corresponding segmentation pattern, with the same area highlighted by red boxes and showed in **(B)**. **(C)** Illustration of the corresponding pixel-level widths for the two modes.

Involuntary eye movements during scanning can cause shifts in the scanning area, leading to linear white noise and artifact streaks (24, 25). Additionally, imaging patterns from different scanning instruments introduce significant noise and artifacts, degrading the quality of retinal vascular imaging (26). As shown in the magnified view in Figure 1, these variations across instruments make it challenging to accurately identify microvascular regions (27, 28). As such, noise and artifacts in the images remain a primary challenge for retinal microvasculature segmentation (29). Additionally, due to the localized nature of convolution operations, deep learning algorithms often struggle to model long-range dependencies, making it hard to capture the detailed retinal microvascular structure (30). Repeated convolution and pooling operations further contribute to semantic information loss (31, 32), resulting in overly large or missing boundaries in microvasculature segmentation (33, 34). Figure 2 illustrates how pixel-level differences lead to oversized segmentation of thicker vascular boundaries, while microvascular structures only 1-2 pixels wide may disappear. Therefore, preserving continuous vascular information is crucial for precise retinal microvasculature segmentation.

To this end, we propose a novel Dual-stream Disentangled Representation Network(D2Net) based on the encoder-decoder architecture, for OCTA retinal vascular segmentation. To further enhance the modeling of vascular structure and noise and artifacts features, we map the extracted high-dimensional features into a latent space for representation. By introducing vascular structure priors, we strengthen the network's capability to represent latent variables effectively. In addition, we construct auxiliary information to enable the dual-stream encoder to focus on different features separately. The optimization procedure is achieved by integrating an image reconstruction term and a Kullback-Leibler (KL) regularization term. The dual-stream encoders focus respectively on learning the noise and artifacts features for image reconstruction as well as learning the latent features of vascular structures for the KL regularization term. This approach enables the disentanglement of vascular structure information and artifacts in OCTA images, thereby making segmentation of retinal microvasculature possible. Subsequently, we develop a novel module to leverage multi-scale contextual information to enhance the perception of continuous vessels, thereby refining segmentation results with better accuracy.

Finally, we conducted detailed and comprehensive manual annotations on OCTA images collected from two different instruments, constructing an internally annotated dataset (FOCA) at the microvascular level to validate the effectiveness of the proposed segmentation method. Additionally, we conducted experiments on four datasets focusing on segmenting varying degrees of vessels, including two public datasets (OCTA-500, ROSE-O) and two private datasets (ROSE-Z, ROSE-H). The experimental results demonstrate that the proposed D2Net achieves superior performance in microvascular segmentation. The contributions are summarized as follows:

• We propose a Dual-stream Disentangled Network (D2Net) capable of learning the representation of vascular structure information and stylistic information in OCTA images, significantly enhancing the model's robustness against noise and artifacts from different instruments.

• We propose a Distance Correlation Energy (DCE) module that utilizes low-dimensional images to construct auxiliary information, ensuring that disentanglement remains stable even when using a single image as input. This approach also enhances the ability of the network to sense the microvascular structure making the segmentation boundary more accurate.

• We extensively evaluate the proposed method on five datasets acquired from multiple OCTA instruments, confirming its state-of-the-art segmentation performance and generalization capabilities.

## 2 Related work

Several works have proposed deep learning methods for OCTA vessel segmentation. To extract more high-level semantic features, Pissas et al. (35) proposed a U-shaped network capable of integrating shallow and deep features. Eladawi et al. (36) introduced an OCT segmentation algorithm based on the Markov Gibbs random field model. Joint-Seg (37) performs joint encoding on OCTA images and utilizes a feature-adaptive filter to provide FAZ and RV-related information for separate decoding branches. These works focus on feature extraction from raw images, which significantly limits the ability to reconstruct and represent microvascular structures. To address the issue of feature degradation caused by convolution and enhance the dependency relationships of contextual features, Chen et al. (30, 38) combined U-Net with Transformer, integrating global attention mechanisms to enhance detailed information and achieve precise localization. Similarly, Wang et al. (39) and Liu et al. (40) propose layer segmentation algorithms specialized for OCT images based on Transformer strategies. Chen et al. (41) combines U-Net and Swin-Uformer to jointly learn global and local information in OCT images, compensating for information loss between layers due to speckle noise.

Despite these advances, attention mechanisms show better performance in compensating for local information, when the focus is on microvascular areas with more refined and complete constraints, the network applies attention enhancement across all regions containing microvessels in OCTA images. This easily leads to boundary overshooting during segmentation of adjacent microvessels, resulting in many false positive predictions, a phenomenon we also observed in our comparative experiments. Recently, Liu et al. (26) proposed a segmentation model that leverages the disentanglement of anatomical and contrast components from paired OCTA images to focus on vascular structure information. However, this method requires a large amount of paired data from different devices for pretraining, increasing the data acquisition threshold. Additionally, it freezes the pre-trained model parameters for supervised learning, which creates limitations in model complexity and portability in the two-stage approach.

OCTANet (4) is a network specifically designed for the segmentation of OCTA vessels with varying thicknesses. It first generates an initial vessel confidence map using a coarse segmentation module based on splitting, and then optimizes the shape of retinal microvessels using a fine segmentation module based on splitting, thereby achieving precise segmentation. VAFF (8) utilizes OCTA images from different layers and incorporates a specialized voting gate mechanism to achieve more accurate vessel localization and segmentation. However, both of these networks tailored for OCTA segmentation tasks are unable to effectively address the artifact and noise interference issues caused by different devices.

The aforementioned methods primarily emphasize combining additional low-level information to improve network segmentation performance, without considering the adverse effects of redundant information reuse and information leakage on network performance. These methods have limitations when learning from the OCTA microvascular regions, as they fail to effectively avoid interference from artifact noise and neglect the utilization of low-dimensional image information from the microvessels.

## 3 Methodology

In this section, we introduce the proposed dual-stream disentangled architecture named D2Net, including vessels extractor, dual-stream disentangled network, distance correlation energy module and loss function for its end-to-end training.

### 3.1 Architecture

**Figure 3:**
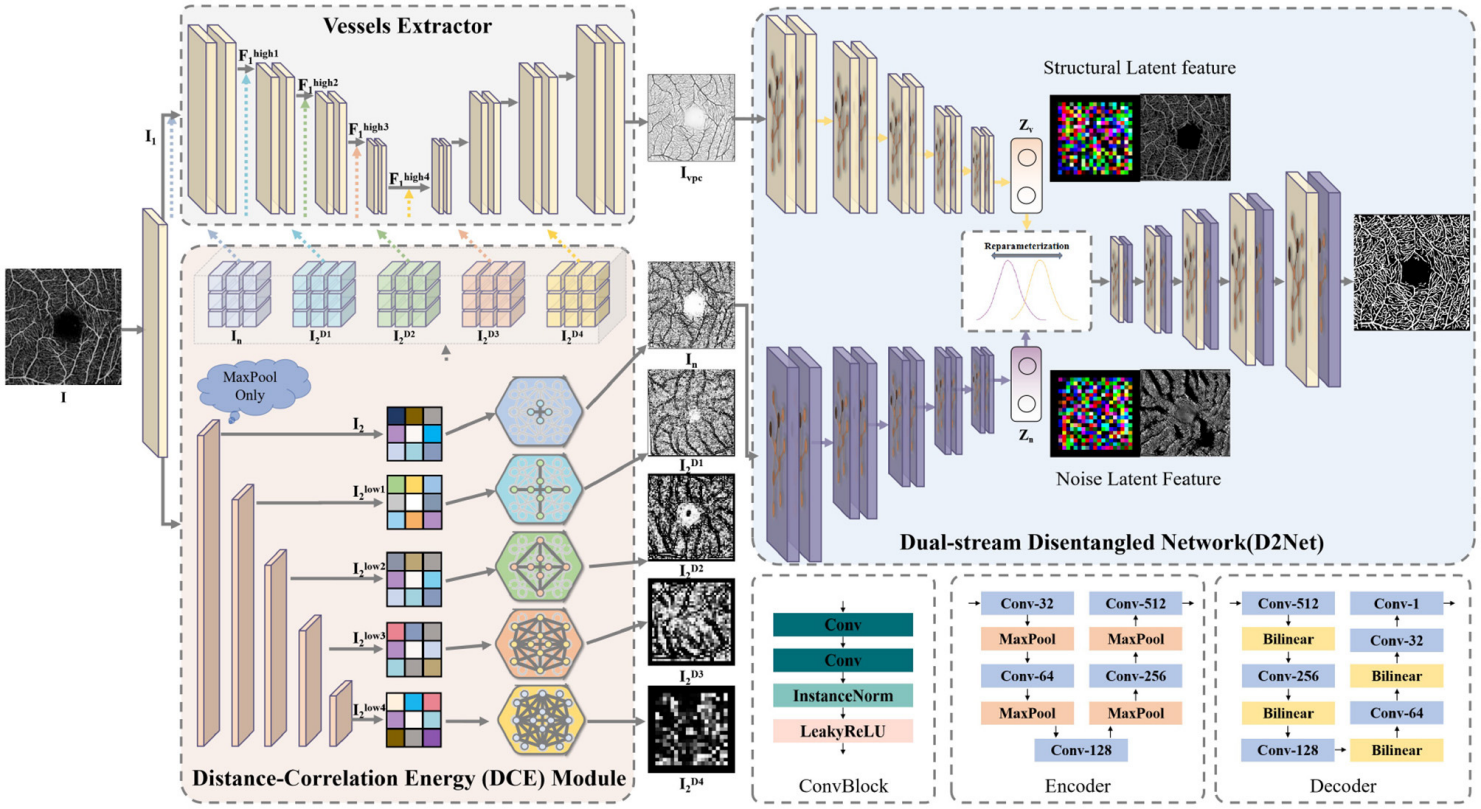
The architecture of our D2Net, consisting of the vessels extractor, dual-stream disentangled network and the distance correlation energy.

The overall architecture of the proposed D2Net is shown in Figure 3. D2Net consists of three main components: the vessels extractor, the dual-stream disentangled network, and the distance correlation energy (DCE) module. The model's goal is to differentiate vascular features from noise and artifacts through disentangled representation learning, reducing interference and improving microvascular segmentation.

The model input is a single-layer *en face* projection from the SVC layer of the OCTA image. The initial OCTA image *I*∈ℝ^*H*×*W*×3^ is duplicated and processed with a 1 × 1 convolutional layer to adjust the channel number to 64, resulting in *I*_1_ and *I*_2_. Subsequently, *I*_1_ and *I*_2_ are sent to the vessels extractor and DCE module, respectively, to obtain a noise-free vascular structure prior *I*_*vpc*_ and a version with noise and artifacts *I*_*n*_. The vessels extractor enhances high-dimensional features at each layer using the DCE module's correlation energy, producing *I*_*vpc*_, which serves as the input for the vascular disentangled stream and provides a clean vascular structure reference. To enable the network to focus on the vascular structure, the second stream must minimize vascular information while highlighting noise and artifacts. Thus, *I*_2_ is enhanced with correlation energy, creating *I*_*n*_, which retains both vascular and noise information, and is used as input for the noise and artifacts disentangled stream.

The vessels extractor in D2Net can be any end-to-end segmentation network; in this paper, a U-shaped architecture is used as the backbone. Notably, D2Net is designed as a plug-and-play framework, allowing any state-of-the-art segmentation network to replace the vessels extractor. The extractor's convolutional block includes two 3 × 3 convolution layers with Instance Normalization and LeakyReLU activation. Maxpooling and bilinear interpolation are used for feature dimension reduction and resolution restoration, respectively. The extractor performs four rounds of feature extraction on *I*_1_, producing feature maps $F_{1}^{high1}$ ($\frac{1}{2}H\times \frac{1}{2}W\times 128$), $F_{1}^{high2}$ ($\frac{1}{4}H\times \frac{1}{4}W\times 256$), $F_{1}^{high3}$ ($\frac{1}{8}H\times \frac{1}{8}W\times 512$), and $F_{1}^{high4}$ ($\frac{1}{16}H\times \frac{1}{16}W\times 512$). For the noise and artifacts disentangled stream, it's only necessary to provide vascular information comparable to the vascular disentangled branch while differing in noise and artifacts. Thus, instead of dimension reduction, the low-dimensional image data *I* is directly enhanced with correlation energy by the DCE module and input into the dual-stream disentangled network.

Each layer in the dual-stream encoder extracts high-level information from two distinct feature types. Specifically, each encoder layer consists of two convolutional layers with instance normalization and LeakyReLU activation, followed by maxpooling for feature extraction. The decoder mirrors this structure, using bilinear interpolation for feature restoration. Additionally, we flatten the extracted latent variables, transform the resulting vector into latent space via linear neurons, reparameterize it, and feed it into the upsampling layer to complete the reconstruction of retinal vessels.

### 3.2 Dual-stream disentangled network

Noise and artifacts can complicate accurate segmentation of microvascular regions. To address this, our dual-stream disentangled network is designed to separate representations of retinal vessel structure from noise and artifact information. This disentanglement, achievable using a single image, facilitates training on more diverse datasets. The dual-stream network includes two encoders with similar structures but independent weights and a shared decoder. Each encoder focuses on specific features: one on the vascular structure and the other on noise and artifacts. With the vascular structure extracted by the vessels extractor as a prior constraint, the network learns to reduce noise interference and achieve more accurate segmentation of microvascular areas. Each encoder maps its input into latent space, where the shared decoder reparameterizes the latent features and adaptively learns feature selection and fusion. The inputs for each stream are tailored: the vascular structure stream uses prior information from the vessels extractor enhanced with multi-scale DCE modules, while the noise and artifacts stream uses OCTA data enhanced with single-scale DCE modules. This setup ensures both streams have consistent vascular structure information during encoding. Given that encoding is a lossy process with a capacity limit (42), the inputs to both streams are designed to maintain identical spatial dimensions.

As shown in Figure 3, in the noise and artifacts stream, *I*2 is enhanced through the DCE module to obtain *I*_*n*_, which increases the distance correlation energy. Subsequently, *I*_*n*_ is then encoded by the noise and artifacts stream encoder, yielding the probability distribution *q*_ϕ_(*Z*_*n*_|*I*_*n*_) in the latent space. The latent feature *Z*_*n*_ has an encoding bottleneck, allowing it to only capture limited information from *I*_*n*_. In the vascular structure stream branch, *I*1 is processed by the vessels extractor and the DCE module, generating *I*_vpc_, which also enhances distance correlation energy. The vascular structure encoder then maps *I*_vpc_ to the probability distribution *q*_ϕ_(*Z*_*v*_|*I*_vpc_) in latent space. Thus, *Z*_*v*_ represents only the vascular structure features, free of noise and artifacts, while *Z*_*n*_ includes similar vascular features alongside unique noise and artifact information. The decoder reconstructs *Z*_*n*_ using the encoding bottleneck to prioritize distinct information while discarding redundancies. Similar to conditional variational autoencoders (43), this process is guided by the evidence lower bound (ELBO):


(1) $$\begin{array}{l} \begin{array}{c} L\left(\theta ,\phi ;I_{n}\right)=E_{q_{\phi }\left(Z_{n}|I_{n}\right)}\left[\log p_{\theta }\left(I_{n}|Z_{n}\right)\right]-\text{KL}\left(q_{\phi }\left(Z_{n}|I_{n}\right)||p\left(Z_{n}\right)\right), \end{array} \end{array}$$

This equation balances the reconstruction likelihood of *I*_*n*_ given *Z*_*n*_ with the KL divergence between the approximate posterior *q*_ϕ_(*Z*_*n*_|*I*_*n*_) and the prior *p*(*Z*_*n*_), ensuring that the network learns to generate accurate reconstructions while maintaining diversity in the latent space.

The encoder maps the input *I*_*n*_ to a probability distribution in the latent space, *q*_ϕ_(*Z*_*n*_|*I*_*n*_), assumed to be a Gaussian distribution $N\left(Z_{n};\mu \left(I_{n}\right),\sigma \left(I_{n}\right)\right)$. The first term, *E*_*q*_ϕ_(*Z*_*n*_|*I*_*n*_)_[log*p*_θ_(*I*_*n*_|*Z*_*n*_)], represents the expected log-likelihood of reconstructing the input data *I*_*n*_ under the latent variable *Z*_*n*_. The decoder samples from the latent variable *Z*_*n*_ and reconstructs the input data *I*_*n*_, denoted as *p*_θ_(*I*_*n*_|*Z*_*n*_). The reconstruction error term involves taking the expectation over the variational posterior distribution *q*_ϕ_(*Z*_*n*_|*I*_*n*_). In practice, this expectation can be computed as ∫*q*_ϕ_(*Z*_*n*_|*I*_*n*_)log*p*_θ_(*I*_*n*_|*Z*_*n*_)*dz*.

This represents the expected log-likelihood of reconstructing *I*_*n*_ under the latent variable *Z*_*n*_. The objective of optimizing the reconstruction error term is to maximize *E*_*q*_ϕ_(*Z*_*n*_|*I*_*n*_)_[log*p*_θ_(*I*_*n*_|*Z*_*n*_)], indicating that we aim for *Z*_*n*_ to accurately reconstruct the input data *I*_*n*_ as closely as possible. This part is akin to minimizing the mean squared error of reconstructing *I*_*n*_ in implementation. The second term *KL*(*q*_ϕ_(*Z*_*n*_|*I*_*n*_)||*p*(*Z*_*n*_)) is the Kullback-Leibler divergence between the variational distribution *q*_ϕ_(*Z*_*n*_|*I*_*n*_) and the prior distribution *p*(*Z*_*n*_) in the latent space, used to regularize the distribution of latent variables.

The decoder parameters are optimized to maximize reconstruction log-likelihood, aiming to capture only the most critical latent features for reconstruction. Due to the limited dimensionality of the latent space, the decoder must prioritize differential information essential for reconstruction. If *Z*_*n*_ were expected to also represent vascular information, it would lead to a significant redundancy between *Z*_*n*_ and *Z*_*v*_ latent features. Thus, the decoder focuses on preserving features in *Z*_*n*_ specific to noise and artifacts, while *Z*_*v*_ retains vascular structure details.

### 3.3 Distance correlation energy module

In OCTA images, accurately segmenting fine microvascular structures is challenging due to complex vascular topology and interference from noise. Convolutional operations alone struggle to capture long-range dependencies and maintain low-dimensional image details, leading to information loss. To address this, we introduce a DCE module to capture multi-scale neighborhood correlations, which enhances segmentation accuracy by improving microvascular representation. The DCE method leverages both low-dimensional neighborhood semantics and high-dimensional feature correlations by constructing an energy matrix that measures pixel correlations. Typically, large differences between neighboring pixels indicate noise or artifacts, while small differences signify continuous vascular structures with higher energy. This helps the network retain vascular structure while minimizing the influence of independent noise.

Specifically, we represent the OCTA *en face* image as an undirected graph *G* = (*V, E*), where *V* represents the set of nodes (vertices) in the graph, and *E* represents the set of edges connecting these nodes. We calculate the distance between each node and its four-connected neighbors, forming a distance correlation matrix *D*. Assuming node *i* has four connected nodes *j*_*p*_ (where *p* = 1, 2, 3, 4), the matrix *D* is defined as:


(2) $$\begin{array}{l} D_{i,j_{p}}=D_{j_{p},i}=||I\left(i\right)-I\left(j_{p}\right)|{|}^{2}, \end{array}$$

where *I*(·) denotes the pixel values from the pre-processed OCTA data *I*_2_ obtained after a 1 × 1 convolution. To address the boundary two-neighbor problem, we introduce additional edges that treat head and tail nodes as independent elements with an infinite distance between them. In this undirected graph, the path distance between nodes is computed by summing the weights of the connecting edges. When structural information in the image is similar, indicating close proximity between two nodes, the network assigns higher correlation weights to that region during training to enhance vascular feature learning. Conversely, regions with greater differences, such as isolated noise, are assigned higher distances to neighboring elements. Instead of discarding these noise-related components entirely, we assign them smaller correlation weights. The distance correlation matrix is then converted into energy weights as supplementary information. *w* can be described as: $w=e^{-\frac{D}{\lambda }}$, where λ is a predefined hyperparameter that adjusts the energy magnitude. Since boundary nodes have infinite distances in the second neighborhood, their energy weights approach zero. This setup enables the network to localize features in images of various sizes while maintaining weak connectivity at boundary nodes. Because features extracted at different layers of the autoencoder capture different levels of semantic information, the DCE module needs to provide energy at multiple scales to the vessels extractor. To ensure alignment, a consistent pooling mechanism is used in the DCE module, matching the perceptual field of the vessels extractor. The DCE module then applies the same number of pooling operations to *I*_2_ as the vessels extractor, representing corresponding low-dimensional signals across scales: $I_{2}^{low1}$ ($\frac{1}{2}H\times \frac{1}{2}W\times 3$), $I_{2}^{low2}$ ($\frac{1}{4}H\times \frac{1}{4}W\times 3$), $I_{2}^{low3}$ ($\frac{1}{8}H\times \frac{1}{8}W\times 3$), and $I_{2}^{low4}$ ($\frac{1}{16}H\times \frac{1}{16}W\times 3$). When features are reduced *k* times, each pixel can represent an energy value within a range of $4\cdot \frac{1}{2^{k}\left(k+1\right)}$. In summary, this approach enhances the semantic boundaries of vessel regions, helping to mitigate annotation errors and improve segmentation model performance for microvascular regions.

### 3.4 Loss function

During network training, standard cross-entropy (CE) loss is computed between the vessel extractor's predictions *Y*_*extract*_ and their corresponding ground truth *Y*_*gt*_ is computed and updated through backpropagation to optimize the network parameters. The CE loss is represented as *L*_*CE*_(*Y*_*extract*_, *Y*_*gt*_). In the dual-stream disentangled network, our aim is to filter out redundant vascular structure information in the noise and artifacts stream. For this purpose, the mean squared error (MSE) loss is used between the reconstructed outputs and the original OCTA image to capture noise and artifacts more effectively. Given the reconstructed output *Y*_rec_ and the image with domain-specific information *Y*_I2_, the MSE loss is defined as *L*_MSE_(*Y*_rec_, *Y*_I2_). To further optimize, KL divergence is employed to measure information loss by comparing the posterior distribution of latent features to a standard normal prior. This approach minimizes information loss in the approximate distribution, prevents distribution shift, and helps maintain the model's generalization capability. This process can be expressed as follows:


(3) $$L_{kl}=KL(q_{\phi }(Z_{n}|I_{n})||p(Z_{n}))=-\frac{1}{2}\sum_{i=1}^{n}\left(1+log\sigma^{2}-\mu^{2}-e^{\sigma }\right),$$

where μ and σ represent the mean and variance of the distribution, respectively.

## 4 Experimental setup

### 4.1 Datasets

In this work, we constructed a new dataset, FOCA, to validate the effectiveness of our proposed method for microvascular segmentation. This dataset specifically focuses on microvascular regions. To further assess the method's robustness across diverse large-vessel segmentation standards, we also conducted experiments on two publicly available datasets (OCTA-500, ROSE-O) and two private datasets (ROSE-Z, ROSE-H).

**FOCA** contains 88 OCTA images from 3 mm × 3 mm SVC scans acquired using the Angiovue (RTVue XR Avanti, Optovue) and Zeiss Cirrus 5000-HD-OCT Angioplex (Carl Zeiss Meditec) instruments. We selected 40 image pairs with optimal imaging quality and aligned them at the pixel level. Each image underwent detailed pixel-level annotation, completed meticulously by a specialist and reviewed independently by another to ensure precision. Referencing OCTA-500 and ROSE-O, we randomized the data, using 32 scans for training and 8 for testing. Additionally, our dataset includes 44 paired, non-annotated images (with completed registration) to facilitate further research into retinal vascular structure using paired OCTA images.

**ROSE-O** includes 39 OCTA images from 3*mm*×3*mm* SVC scans obtained using the RTVue XR Avanti SD-OCT (Optovue, USA). We followed the same train-test split used in (4) (30 scans for training, 9 for testing), utilizing both the scans and their manual delineations.

**ROSE-Z** consists of 126 OCTA images from the Zeiss Cirrus 5000-HD-OCT Angioplex (Carl Zeiss Meditec) instrument. This dataset includes enface SVC, DVC, and IVC layer images from 42 subjects, encompassing 15 cases of diabetic retinopathy, 2 of Alzheimer's disease, and 25 healthy controls. We selected the 3*mm*×3*mm* SVC layer scans and their manual annotations for our experiments.

**ROSE-H** includes 60 OCTA images obtained using the Heidelberg Spectralis OCT2 (Heidelberg Engineering, Germany) instrument, with 3*mm*×3*mm* SVC layer scans and corresponding manual annotations. This dataset comprises 8 images with choroidal neovascularization and 12 from healthy controls. Following the training strategy in (8), we trained using the ROSE-O dataset's training subset and reserved ROSE-H solely for testing to evaluate generalizability across OCTA images from different instruments.

**OCTA-500** contains 200 OCTA images (No. 10301−No. 10500), collected with a 70 kHz SD-OCT (RTVue-XR, Optovue, CA). The scans, centered on the macula with a 3*mm*×3*mm* range, include manual annotations. We adhered to the train-validation-test split in (44): images No. 10301−10440 for training, No. 10441−10450 for validation, and No. 10451−10500 for testing.

### 4.2 Implementation details

The proposed D2Net was implemented using the PyTorch framework and trained on a single NVIDIA GeForce GTX 3090Ti with 24GB of memory. We used the Adam optimizer with an initial learning rate of 0.0005 and a batch size of 4. The model was trained for 200 epochs, with all images resized to 512 × 512 pixels. No learning rate decay was applied. All experiments were independently validated, and metrics were not directly referenced. To ensure a fair comparison, the same parameter settings and training strategies were applied to all comparison methods. During training, all models underwent the same augmentation techniques, including random horizontal and vertical flips, random rotations, and random cropping.

### 4.3 Evaluation metrics

To comprehensively and objectively evaluate the segmentation performance of the proposed methods, the following metrics were calculated between the segmentation results and manual delineations for each model:

- Area Under the ROC Curve (AUC);
- Accuracy (ACC) = (TP + TN)/(TP + TN + FP + FN);
- Dice coefficient (Dice) = 2 × TP/(FP + FN + 2 × TP);
- G-mean score(GMEAN) = $\sqrt{\text{sensitivity}\times \text{specificity}}$;

where TP, TN, FP, and FN represent True Positives, True Negatives, False Positives, and False Negatives, respectively. Sensitivity (also known as recall or true positive rate) is the proportion of actual positives correctly identified by the model, calculated as TP/(TP + FN). Specificity is the proportion of actual negatives correctly identified, calculated as TN/(TN + FP). The GMEAN score balances sensitivity and specificity, offering a more holistic measure of a model's performance in binary classification tasks.

## 5 Experimental results

Since retinal vascular region segmentation is a dense prediction task, we evaluated the performance of our model by comparing it with several recent methods in medical image segmentation, including UNet (35), UNet++ (45), CENet (33), CSNet (10), OCTANet (4), VAFF (8), SwinUNet (46), TransUNet (30), and UTNet (38). To ensure a fair comparison, all methods used the same training approach and optimization strategy, as described in Section 4.2.

### 5.1 Subjective comparisons

#### 5.1.1 Qualitative comparisons of annotation and segmentation

**Figure 4:**
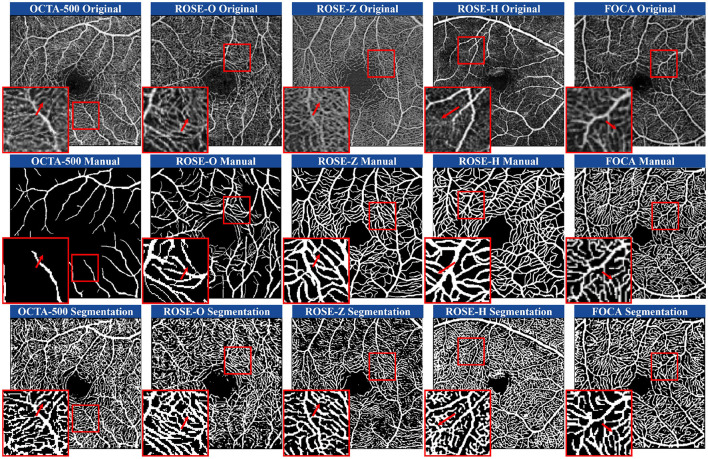
Comparison of manual annotations and microvascular region prediction results across four heterogeneous datasets and one homogeneous dataset. The prediction model was trained using D2Net on the FOCA dataset.

Figure 1 shows examples of manual annotations from the OCTA-500, ROSE-O, ROSE-Z, and ROSE-H datasets. In many cases, the manual annotations do not accurately match the actual microvascular regions, and these regions are often overlooked during annotation. In Figure 4, we use the microvascular region annotations from the FOCA dataset to guide segmentation, leading to more accurate and refined results across the four datasets. For example, in the OCTA-500 segmentation results, the large vessels in the original image and the predictions are mostly consistent, but the manual annotations for large vessels near the FAZ region are incomplete. Additionally, the segmentation of microvascular regions is much more accurate compared to the manual annotations, which often fail to capture these regions. In the ROSE-O and ROSE-Z datasets, the predictions are more refined and accurate.

In the generalization experiments on the ROSE-H dataset, while the continuity of capillaries was not as well-preserved as in the other domains, the predictions for large vessels remained accurate. In areas with noise and artifact stripes, manual annotations mistakenly identify them as vessels, while our model correctly classifies them as non-vessel structures. Our method shows a stronger ability to predict low-contrast regions, generating visually richer and usually accurate microvascular predictions. We also conducted tests on the same-domain FOCA dataset. In these examples, we selected lower-contrast original images for demonstration. Our method outperforms in boundary handling, accurately matching the boundary width of professional manual annotations. Furthermore, in blurry microvascular regions, the predicted results are generally complete and accurate.

#### 5.1.2 Qualitative comparisons with state-of-the-art methods

**Figure 5:**
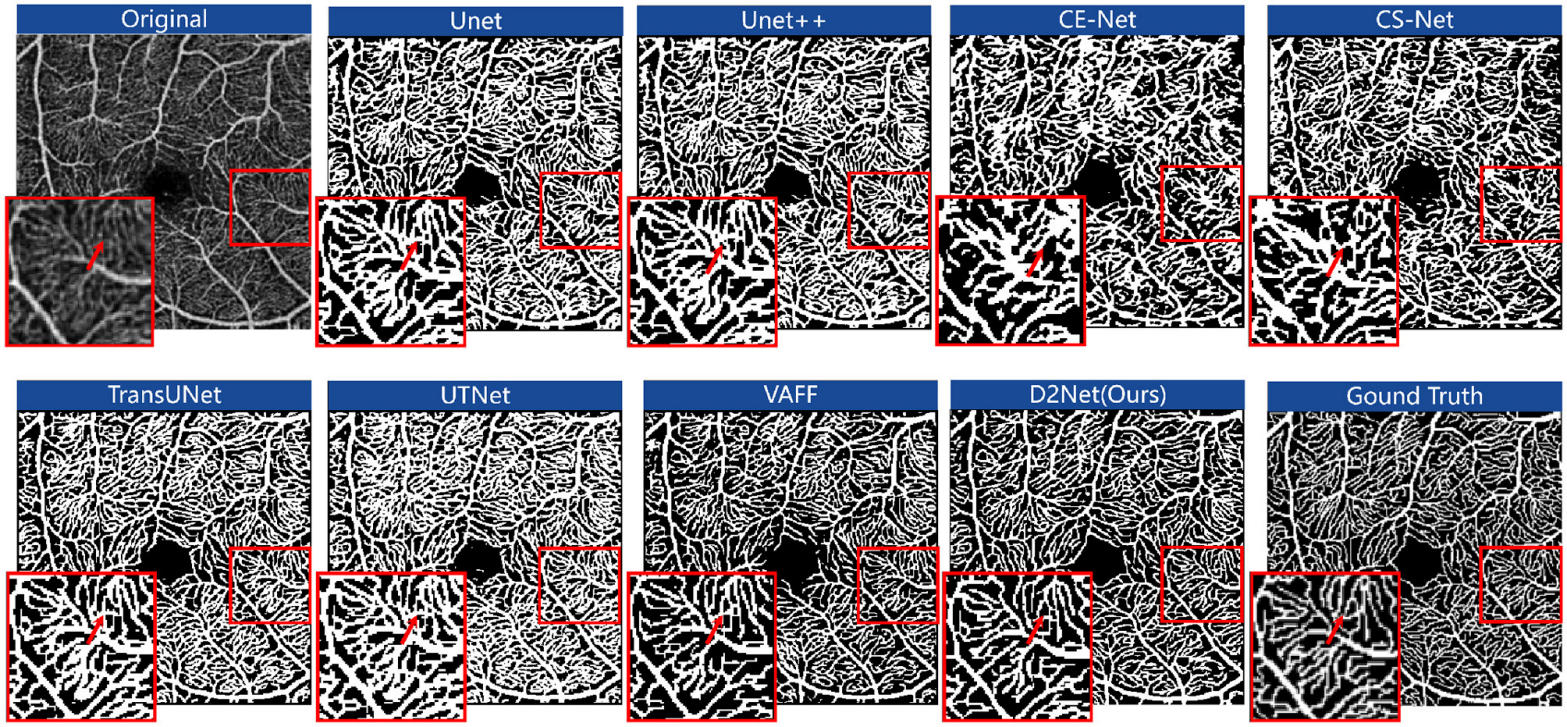
The segmentation results of OCTA images for different methods on the FOCA dataset. The results are shown in red boxes as zoomed-in examples of the segmentation details.

Figure 5 shows the segmentation results of OCTA images from the FOCA dataset using different methods. D2Net outperforms Unet, Unet++, CE-Net, and CS-Net in boundary segmentation. For example, in the red-highlighted areas, these methods over-segment large vessels and microvasculature, extending beyond the vessel boundaries. Due to fine annotations and the strong memory capabilities of these networks, continuous microvascular regions are often over-segmented and identified as block-like areas, losing their microvascular shape. TransUNet and UTNet focus more on local microvascular regions, showing better performance in these areas, but they still exhibit the issue of segmentation extending beyond vessel boundaries. While the over-segmentation problem has improved compared to the other methods, it remains notable. VAFF's voting mechanism helps prevent the network from forcibly memorizing and stacking results, providing better boundary handling. However, it struggles with learning microvascular regions. For example, in the FAZ region, microvascular predictions are inaccurate, with many breakages and incorrect segmentations. In contrast, our approach shows a trend of reduced over-segmentation and under-segmentation. This improvement is due to the DCE module, which effectively uses the correlation energy information of neighboring pixels to assign higher weights to continuous vessels and capture long-distance information.

### 5.2 Quantitative comparisons

#### 5.2.1 Quantitative comparison of microvascular datasets

To evaluate the effectiveness of our method for retinal microvascular segmentation, we conducted quantitative experiments using the FOCA dataset, which focuses on microvascular regions. The results, shown in Table 1, demonstrate that our method, D2Net, outperforms existing state-of-the-art approaches. Specifically, D2Net achieved the best segmentation performance across multiple key metrics, including AUC, ACC, GMEAN, and DICE. As indicated in the table, D2Net leads in all metrics, with a Dice score 2.01 percentage points higher than the second-best method. This highlights the effectiveness of our dual-stream disentangled network, which reduces noise and artifact interference by separately learning vascular structures and image noise, leading to more accurate and complete segmentation results.

**Table 1:** Comparison of microvascular segmentation performance of different segmentation methods on the dataset FOCA.

**Method**	**AUC (%)**	**ACC (%)**	**GMEAN (%)**	**DICE (%)**
CENet	78.09 ± 5.54	68.19 ± 6.74	69.92 ± 5.74	60.21 ± 6.44
CSNet	80.02 ± 7.45	71.19 ± 7.53	72.06 ± 7.45	61.06 ± 8.88
SwinUNet	78.60 ± 6.52	67.34 ± 6.38	70.28 ± 6.48	60.40 ± 7.38
TransUNet	78.61 ± 6.55	71.57 ± 6.12	70.55 ± 6.68	60.09 ± 8.47
Unet	79.69 ± 7.51	71.34 ± 8.09	70.77 ± 7.54	60.66 ± 9.12
Unet++	80.19 ± 7.28	71.48 ± 6.93	71.85 ± 7.23	60.99 ± 8.75
UTNet	75.96 ± 6.24	69.06 ± 5.92	69.15 ± 6.23	58.55 ± 7.53
OCTANet	73.26 ± 5.45	70.98 ± 6.21	71.61 ± 4.11	61.47 ± 4.97
VAFF	79.01 ± 6.73	70.48 ± 6.62	71.16 ± 6.69	61.01 ± 8.13
Ours	81.14 ± 7.48	72.03 ± 8.08	72.90 ± 7.55	63.48 ± 8.88

#### 5.2.2 Quantitative comparison of large vascular datasets

We conducted quantitative comparisons across four large vessel datasets to demonstrate the the superior performance of our proposed method. All comparison methods were independently validated in our experimental environment. Table 2 presents the segmentation results of D2Net on the public datasets OCTA-500 and ROSE-O, while Table 3 shows results on the private datasets ROSE-Z and ROSE-H. As seen in both tables, D2Net outperforms other methods in all metrics. It accurately captures large vessels with high precision and consistency, and performs excellently in both DICE and GMEAN, showing its ability to identify large vessel regions while avoiding under-segmentation. These results highlight D2Net's superior performance in large vessel segmentation tasks. Furthermore, we assessed the model's generalization performance, with results from the cross-domain dataset ROSE-H (not used in training) shown in Table 3. The results demonstrate that D2Net not only generalizes well but also achieves the best performance compared to other methods.

**Table 2:** Comparison of large vessel segmentation results between different methods on the public datasets ROSE-O and OCTA-500.

**Method**	**ROSE-O**				**OCTA-500**			
	**AUC (%)**	**ACC (%)**	**GMEAN (%)**	**DICE (%)**	**AUC (%)**	**ACC (%)**	**GMEAN (%)**	**DICE (%)**
CENet	82.34 ± 0.87	90.61 ± 1.02	81.22 ± 1.01	73.98 ± 1.08	99.56 ± 7.39	98.31 ± 1.45	93.38 ± 7.87	87.50 ± 0.69
CSNet	85.37 ± 0.78	89.53 ± 0.81	**85.10** **±0.83**	74.25 ± 0.65	98.89 ± 0.95	98.69 ± 0.17	95.93 ± 0.98	90.50 ± 1.05
SwinUNet	80.62 ± 0.98	82.59 ± 2.06	80.54 ± 0.93	63.17 ± 1.20	98.13 ± 2.05	96.22 ± 0.37	89.95 ± 0.87	90.45 ± 1.25
TransUNet	83.67 ± 1.00	87.10 ± 1.11	83.48 ± 1.03	69.94 ± 0.94	99.60 ± 1.10	98.39 ± 0.20	96.65 ± 1.13	88.77 ± 1.28
UNet	85.17 ± 0.88	88.75 ± 0.90	84.96 ± 0.91	73.04 ± 0.63	98.59 ± 0.96	98.47 ± 0.22	96.81 ± 0.99	89.31 ± 1.47
UNet++	84.27 ± 0.75	87.74 ± 1.49	84.07 ± 0.74	71.22 ± 0.92	99.67 ± 0.73	97.48 ± 0.29	96.46 ± 0.73	88.89 ± 1.54
UTNet	79.82 ± 3.25	82.68 ± 3.76	79.61 ± 3.34	62.01 ± 2.81	99.69 ± 1.54	98.59 ± 0.25	94.42 ± 1.64	89.43 ± 2.28
OCTANet	85.26 ± 0.84	89.58 ± 0.98	84.97 ± 0.88	74.27 ± 0.89	96.45 ± 0.74	98.69 ± 0.17	96.41 ± 0.76	90.49 ± 0.98
VAFF	81.52 ± 0.87	90.30 ± 0.85	79.95 ± 1.05	74.36 ± 1.12	99.66 ± 1.16	98.67 ± 0.19	95.43 ± 1.22	90.30 ± 1.37
Ours	87.71 ± 0.95	90.96 ± 0.84	82.87 ± 1.09	75.37 ± 1.11	99.82 ± 0.77	98.75 ± 0.16	96.96 ± 0.80	91.23 ± 0.08

**Table 3:** Comparison of large vessel segmentation results between different methods on the private dataset ROSE-Z and ROSE-H.

**Method**	**ROSE-Z**				**ROSE-H**			
	**AUC (%)**	**ACC (%)**	**GMEAN (%)**	**DICE (%)**	**AUC (%)**	**ACC (%)**	**GMEAN (%)**	**DICE (%)**
CENet	79.42 ± 0.25	86.66 ± 0.70	77.79 ± 0.41	72.32 ± 0.32	81.31 ± 1.09	89.13 ± 0.92	79.96 ± 1.30	74.16 ± 1.32
CSNet	84.89 ± 0.66	85.97 ± 1.28	84.85 ± 0.64	75.89 ± 1.47	80.74 ± 1.16	89.63 ± 0.75	78.98 ± 1.45	74.27 ± 1.47
SwinUNet	80.54 ± 0.74	81.44 ± 1.11	80.51 ± 0.73	69.95 ± 1.17	81.56 ± 0.51	84.89 ± 1.52	81.27 ± 0.66	70.08 ± 0.49
TransUNet	84.49 ± 0.82	84.27 ± 1.12	84.47 ± 0.82	74.83 ± 1.23	82.59 ± 0.69	87.78 ± 0.78	81.25 ± 0.75	72.70 ± 1.22
UNet	84.60 ± 0.71	86.50 ± 0.97	84.49 ± 0.70	75.61 ± 1.25	82.84 ± 0.65	89.42 ± 0.93	80.80 ± 0.64	74.58 ± 1.45
UNet++	84.88 ± 0.87	85.37 ± 1.13	84.87 ± 0.86	75.91 ± 1.37	82.35 ± 1.08	87.69 ± 1.23	71.74 ± 1.14	73.38 ± 1.29
UTNet	81.04 ± 1.92	83.91 ± 3.02	80.66 ± 2.01	72.26 ± 2.15	79.82 ± 1.88	85.73 ± 2.27	78.81 ± 2.64	69.63 ± 1.75
OCTANet	84.28 ± 0.65	85.69 ± 0.76	84.22 ± 0.65	75.71 ± 0.98	81.99 ± 1.14	85.70 ± 0.94	80.69 ± 1.34	74.42 ± 1.44
VAFF	84.29 ± 0.40	87.09 ± 0.75	84.05 ± 0.40	75.96 ± 0.82	82.45 ± 0.96	89.56 ± 0.81	81.35 ± 1.13	74.61 ± 1.04
Ours	85.58 ± 0.38	87.77 ± 0.76	85.28 ± 0.39	77.72 ± 0.81	83.83 ± 1.08	90.67 ± 0.86	82.73 ± 1.26	75.97 ± 1.24

## 6 Discussion

### 6.1 Ablation studies

**Figure 6:**
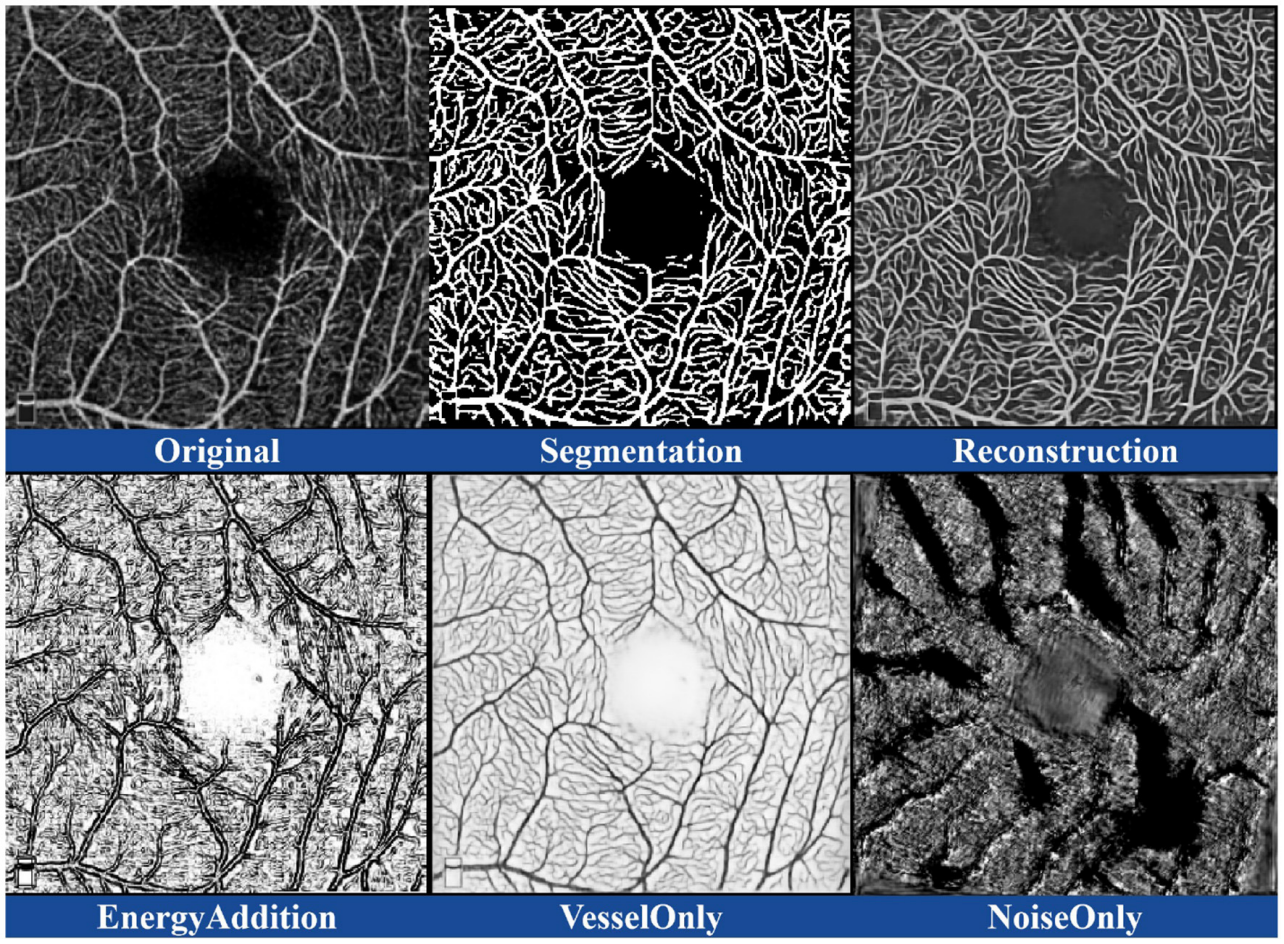
Illustration of the visual representation of various ablation results.

To validate the effectiveness of the dual-stream disentangled network and the DCE module in accurately segmenting retinal microvascular structures, we used a U-shaped architecture as the backbone for the retinal vessel extractor on the OCTA-500 dataset. We performed ablation experiments with two versions: D2Net without the DCE module and D2Net with the DCE module. The results are summarized in Table 4, and the visual effects are shown in Figure 6. Specifically, *Original, Segmentation*, and *Reconstruction* show the original OCTA image, segmentation, and reconstruction results, respectively. *EnergyAddition* demonstrates the intensity patterns after adding the DCE module, while *VesselOnly* and *NoiseOnly* display the disentangled outputs focusing on vascular structures and noise, respectively.

**Table 4:** Ablation results for D2Net and DCE module.

**Method**	**AUC**	**ACC**	**GMEAN**	**DICE**
BaseLine	99.11 ± 0.74	98.28 ± 0.17	96.45 ± 0.77	89.30 ± 0.95
BaseLine+DCE	99.17 ± 0.81	98.70 ± 0.17	96.09 ± 0.82	90.48 ± 1.01
BaseLine+D2Net	99.19 ± 0.86	98.79 ± 0.18	96.14 ± 0.89	90.31 ± 1.01
BaseLine+D2Net+DCE	99.94 ± 0.77	98.94 ± 0.16	96.76 ± 0.80	91.43 ± 0.88

The results highlight the effectiveness of the ablated components, showing that the complete version of D2Net significantly improves various evaluation metrics. This enhancement is due to the effective integration of key components in our method. First, the dual-stream disentangled network in D2Net plays a crucial role in improving segmentation accuracy by better distinguishing between noise and actual vessel structures, reducing the likelihood of missegmentation. Baseline methods struggle to make this distinction. Additionally, the DCE module enhances segmentation by utilizing low-dimensional image neighborhood energy, which improves vessel morphology and topology representation. This helps prevent segmentation errors caused by a lack of contextual information. In summary, D2Net leverages the synergistic effects of the dual-stream disentangled network and DCE module to reduce noise and artifact interference, improving the detection of continuous vascular structures and boosting overall performance. These results demonstrate the superior performance, robustness, and accuracy of our method in retinal microvascular segmentation, even in noisy environments. The proposed dual-stream disentangled network is highly portable and requires minimal data acquisition. It can be easily integrated into any end-to-end extractor, needing only access to the target of interest for the task. In our structure stream branch, the encoding is designed to capture specific information, allowing the decoder to efficiently filter out irrelevant details during image reconstruction in the other branch.

### 6.2 Comparison of real data

**Figure 7:**
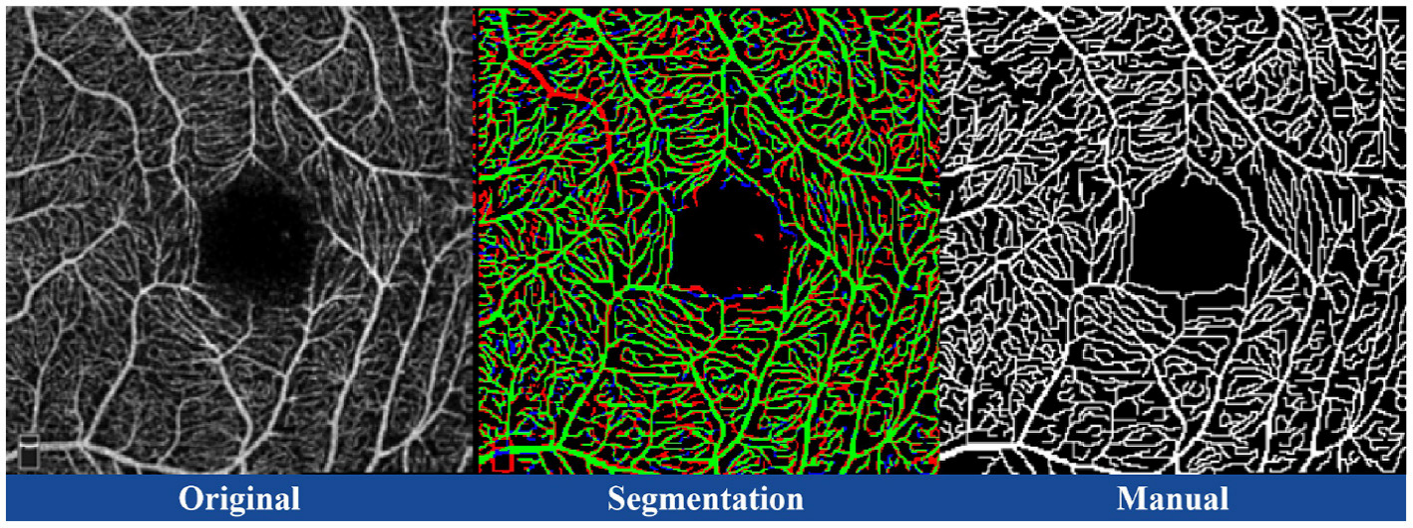
Illustration of the original image corresponding to the FOCA dataset, segmentation result, and manual annotation. In the segmentation result, green represents True Positives (TP), black represents True Negatives (TN), red represents False Positives (FP), and blue represents False Negatives (FN).

D2Net effectively learns vascular structures while decoupling OCTA vascular features from image noise and artifacts. As shown in Table 1, decoupling representation reduces interference from noise and artifacts, enhancing the model's ability to learn vascular structures. The VesselOnly subplot in Figure 7 demonstrates that the network extracts vascular structures, particularly in microvascular regions, more accurately.

As explained in Section 5.1, our model understands vascular structure information rather than relying on memorized answers to make final predictions. To demonstrate the network's error-correction ability, we selected an image from the FOCA dataset with obvious mislabeling for comparison (Figure 7). By calculating the TP, TN, FP, and FN metrics for segmentation and manual annotations, we visualized them using different colors. A continuous large vascular structure in the upper left of the image was confidently identified by the segmentation, which was missed in the manual annotation.

### 6.3 Microvascular morphometric measurements and clinical disease analysis

The study of retinal microvascular changes has gained significant attention, as these changes are closely linked to cognitive impairment. Abnormalities in vascular structures are associated with neurodegenerative diseases such as Alzheimer's disease (AD) and mild cognitive impairment (MCI) (47). This study explores how retinal microvascular morphology differs between AD, MCI, and healthy control (Control) groups to better understand how cognitive impairment stages affect microvascular changes. We evaluate these changes using five metrics: vascular tortuosity (VT), vascular density (VAD), vascular length density (VLD), vessel bifurcation number (VBN), and vascular fractal dimension (VFD).

- VT: Measures the tortuosity of the microvasculature.
- VAD: Measures the total length of perfused retinal microvasculature per square millimeter.
- VLD: Measures the ratio of the number of microvascular centerline pixels to the area of the analyzed region.
- VBN: Counts the vessel bifurcation points.
- VFD: Measures the geometric complexity of the microvasculature.

We applied D2Net to segment 90 OCTA images from AD, MCI, and Control groups (30 images per group), trained on the full FOCA dataset. The metrics VT, VAD, VLD, VBN, and VFD were measured at the SVC layer of the OCTA images, with results shown in Table 5. VT showed a slight decrease in AD and MCI compared to Control, though the difference was not statistically significant (*p*>0.05), suggesting minimal changes in tortuosity in early cognitive impairment. However, both VAD and VLD decreased significantly in AD and MCI compared to Control (*p* < 0.01), indicating reduced blood supply and a sparse microvascular structure as cognitive impairment progresses. VBN increased in AD and MCI, while it was lower in Control (*p* < 0.01), suggesting vascular remodeling to compensate for ischemic conditions. Finally, VFD slightly decreased in AD and MCI (*p* < 0.01), consistent with studies showing reduced geometric complexity in the microvasculature as cognitive impairment progresses.

**Table 5:** Comparisons of OCTA measurement metrics between healthy control, MCI, and AD participants were conducted, with *p*-values obtained through ANOVA.

**Metrics**	**AD**	**MCI**	**Control**	***p***
VT	1.51 (0.14)	1.50 (0.15)	1.58 (0.10)	*p*>0.05
VAD	21.75 (3.00)	20.44 (3.01)	21.97 (2.59)	*p* < 0.01
VLD	7.99 (1.13)	7.43 (1.19)	8.27 (0.86)	*p* < 0.01
VBN	250 (70)	256 (76)	238 (62)	*p* < 0.01
VFD	1.54 (0.03)	1.53 (0.04)	1.55 (0.02)	*p* < 0.01

**Figure 8:**
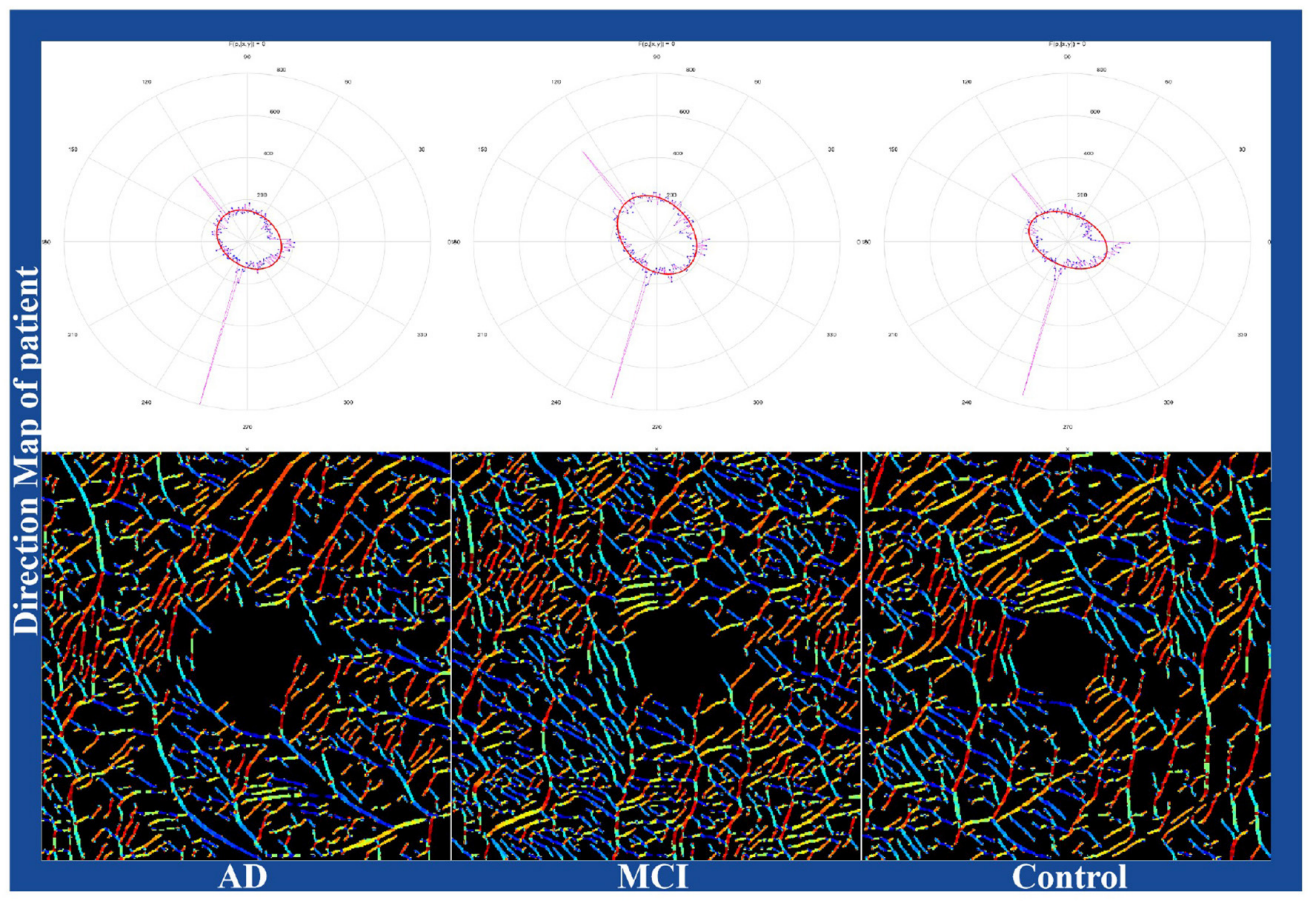
Visualization of direction maps of blood flow signals. The same color represents the same blood flow direction.

We also visualized these metrics as blood flow direction maps, shown in Figure 8. Due to space limitations, results for two subjects from each group are displayed. These findings suggest that retinal microvascular changes are closely linked to cognitive impairment stages. In particular, the significant changes in VAD, VLD, VBN, and VFD emphasize their potential for distinguishing AD and MCI patients from healthy controls. Future research should explore how these metrics can be used to track cognitive impairment progression and therapeutic effectiveness.

### 6.4 Limitations

Our work highlights the limitations of previous microvascular segmentation tasks, which rely on detailed information from small field-of-view (FOV) scans. During our experiments, we found that D2Net consistently performed best in cross-domain data prediction. This led us to explore segmentation of images scanned across different visual fields. However, we discovered significant discrepancies between vascular structures in small and large FOVs. In cross-field segmentation, noise and artifacts are often misidentified as vascular structures, leading to over-segmentation. Moving forward, we plan to further investigate cross-domain and large-field segmentation tasks in medical imaging, with a focus on disentangled segmentation across different domains.

## 7 Conclusion

We have developed a dual-stream disentangled representation network (D2Net) for OCTA retinal microvascular segmentation. The network effectively separates vascular structures from noise and artifacts, improving segmentation accuracy, especially in microvascular regions. The DCE module enhances vessel distance correlation, capturing low-dimensional information often lost during convolution and pooling. Experimental results show that D2Net reduces interference from noise and artifacts, performs well in microvascular and larger vascular region segmentation, and demonstrates strong generalizability. Additionally, statistical analysis of retinal morphology measurements for Control, MCI, and AD patients suggests that D2Net supports clinical diagnosis.

## Data Availability

The original contributions presented in the study are included in the article/supplementary material, further inquiries can be directed to the corresponding authors.
